# The statistical importance of P-POSSUM scores for predicting mortality after emergency laparotomy in geriatric patients

**DOI:** 10.1186/s12911-020-1100-9

**Published:** 2020-05-07

**Authors:** Yang Cao, Gary A. Bass, Rebecka Ahl, Arvid Pourlotfi, Håkan Geijer, Scott Montgomery, Shahin Mohseni

**Affiliations:** 1 Clinical Epidemiology and Biostatistics, School of Medical Sciences, Örebro University, 70182 Örebro, Sweden; 2 Faculty of Medicine and Health, School of Medical Sciences, Department of Surgery, Örebro University, Örebro, Sweden; 3 Department of Surgery, Tallaght University Hospital, Dublin, Ireland; 4 Department of General Surgery, Karolinska University Hospital, Stockholm, Sweden; 5 Department of General Surgery, Örebro University Hospital, Örebro, Sweden; 6 Department of Radiology, Faculty of Medicine and Health, Örebro University, Örebro, Sweden; 7 Clinical Epidemiology Division, Department of Medicine, Karolinska Institutet, 17177 Stockholm, Sweden; 8 Department of Epidemiology and Public Health, University College London, London, WC1E 6BT UK

**Keywords:** P-POSSUM, Emergency laparotomy, Geriatric, Machine learning, Prediction, Permutation variable importance, Gini importance

## Abstract

**Background:**

Geriatric patients frequently undergo emergency general surgery and accrue a greater risk of postoperative complications and fatal outcomes than the general population. It is highly relevant to develop the most appropriate care measures and to guide patient-centered decision-making around end-of-life care.

Portsmouth - Physiological and Operative Severity Score for the enumeration of Mortality and morbidity (P-POSSUM) has been used to predict mortality in patients undergoing different types of surgery. In the present study, we aimed to evaluate the relative importance of the P-POSSUM score for predicting 90-day mortality in the elderly subjected to emergency laparotomy from statistical aspects.

**Methods:**

One hundred and fifty-seven geriatric patients aged ≥65 years undergoing emergency laparotomy between January 1st, 2015 and December 31st, 2016 were included in the study. Mortality and 27 other patient characteristics were retrieved from the computerized records of Örebro University Hospital in Örebro, Sweden. Two supervised classification machine methods (logistic regression and random forest) were used to predict the 90-day mortality risk. Three scalers (Standard scaler, Robust scaler and Min-Max scaler) were used for variable engineering. The performance of the models was evaluated using accuracy, sensitivity, specificity and area under the receiver operating characteristic curve (AUC). Importance of the predictors were evaluated using permutation variable importance and Gini importance.

**Results:**

The mean age of the included patients was 75.4 years (standard deviation =7.3 years) and the 90-day mortality rate was 29.3%. The most common indication for surgery was bowel obstruction occurring in 92 (58.6%) patients. Types of post-operative complications ranged between 7.0–36.9% with infection being the most common type. Both the logistic regression and random forest models showed satisfactory performance for predicting 90-day mortality risk in geriatric patients after emergency laparotomy, with AUCs of 0.88 and 0.93, respectively. Both models had an accuracy > 0.8 and a specificity ≥0.9. P-POSSUM had the greatest relative importance for predicting 90-day mortality in the logistic regression model and was the fifth important predictor in the random forest model. No notable change was found in sensitivity analysis using different variable engineering methods with P-POSSUM being among the five most accurate variables for mortality prediction.

**Conclusion:**

P-POSSUM is important for predicting 90-day mortality after emergency laparotomy in geriatric patients. The logistic regression model and random forest model may have an accuracy of > 0.8 and an AUC around 0.9 for predicting 90-day mortality. Further validation of the variables’ importance and the models’ robustness is needed by use of larger dataset.

## Background

Healthcare services worldwide are challenged by an aging population. In most developed countries, the geriatric population is the fastest-growing group [1]. These geriatric patients frequently undergo emergency general surgery and accrue a greater risk of postoperative complications and fatal outcomes than the general population. Patients over the age of 70 years face a 30-day mortality risk of over 20% following emergency laparotomy and the risk rises sustainably after 80 years [2, 3]. The risk increases for every decade after 60 years of age with a predicted survival less than 10% in patients over 90 years of age [4]. It is therefore highly relevant to validate robust outcome predictors in this patient group in order to develop the most appropriate care measures and to guide patient-centered decision-making around end-of-life care.

Historically, several composite scoring instruments have been developed to predict mortality of the surgical patient including POSSUM (Physiological and Operative Severity Score for the enumeration of Mortality and morbidity) [5] and its modified version P-POSSUM (Portsmouth - POSSUM) [6], SRS (Surgical Risk Scale) [7], PMP (Pre-operative Mortality Predictor) [8], ASA (American Society of Anaesthesiology) classification [9], the National Emergency Laparotomy Audit (NELA) risk model [10], APACHE (Acute Physiology and Chronic Health Evaluation) [11] and physical frailty as measured through osteopenia and sarcopenia [12, 13]. While previous studies have demonstrated both strengths and weaknesses in these instruments, there is no consensus as to which one is more reliable in the geriatric population subjected to emergency laparotomy or how the instruments compare to each other in their ability to predict mortality [14]. P-POSSUM has been used to predict mortality in neurosurgical patients undergoing craniotomy [15], gastric cancer patients [16, 17], patients in level 1 critical care setting [18], after oesophagogastric resections [19], and patients undergoing emergency laparotomy [20], however, its predictive ability is diverse among the different clinical settings. Few studies have investigated the ability of P-POSSUM to predict mortality risk in the elderly after surgery, and these studies suggest that P-POSSUM scoring may be a valid predictor with moderate discrimination, however, its relative importance in the prediction model, compared to other predictors, is yet to be determined [13, 21]. In a previous study, the authors assessed the associative performance of P-POSSUM and other predictors of frailty in calculating 90-day mortality for geriatric patients undergoing emergency laparotomy. These results suggest that a greater emphasis on ASA classification, age, surgical indication and procedure as well as packed red blood cell (PRBC) transfusion requirements and admission systolic blood pressure in modification of the P-POSSUM score may be required to achieve a strong predictive power in this population [22]. We hypothesized that P-POSSUM scores may significantly increase the predictive ability of the statistical models for predicting mortality in geriatric patients after emergency laparotomy. The aim of the current study was to assess the relative importance of readily available patient demographic and clinical characteristics, osteopenia as a surrogate measure for frailty, and P-POSSUM scores for predicting the mortality risk, rather than to interpret the associations between the variables and the mortality.

## Methods

### Patients and variables

Ethical approval for this study was obtained from the institutional review board of Uppsala County (Ref. 2017/421). All geriatric patients (≥ 65 years) undergoing emergency laparotomy between January 1st, 2015 and December 31st, 2016 were included in the study. Patients who had a conversion from laparoscopic surgery to laparotomy or those subjected to laparotomy due to traumatic injury were not included in the studied cohort. Patients’ characteristics (or features in terms of data science), including age, sex, body mass index (BMI), diagnosis according to the International Statistical Classification of Diseases (ICD) 10th version, admission blood tests and vital signs, transfusion requirements during the hospital stay, Charlson Comorbidity Index (CCI) score, ASA classification, reason for operation and surgical procedure performed, post-operative complications, osteopenia, sarcopenia, P-POSSUM, and 90-day mortality, were retrieved from the computerized records of Örebro University Hospital in Örebro, Sweden.

Osteopenia and sarcopenia were assessed by a consultant radiologist using the most recent (≤ 90 days prior to surgery) computed tomography (CT) for measurements of bone density and muscle area. If both a low-dose and a normal-dose radiation CT were performed within 90 days before surgery, the normal-dose study was chosen. Sarcopenia was measured as total skeletal muscle area in a transaxial CT slice, 3 or 5 mm thick, at the L3 vertebral level. Details of the method has been described elsewhere [22]. P-POSSUM scores were calculated using the equation below, which is a combination of 12 weighted physiological and six operative variables obtained for individual patients [5, 6, 15]:

$$ \mathit{\ln}\ \left[R/\left(1-R\right)\right]=-9.37+0.19\times physiological\kern0.4em score+0.15\times operative\kern0.4em score $$

where the constituent variables of the physiological score operative variables are orderly graded as 1, 2, 4 or 8 based on their magnitude then summated to form a physiological score and operative severity score.

### Predictive models and validation

The widely-used machine-learning method for supervised classification problems, logistic regression (LR), was used for predicting the mortality in 90 days after emergency laparotomy in the specified geriatric patient group. Its performance was also compared to the random forest (RF) algorithm, another conventional machine method, which calculates Gini importance or mean decrease in impurity (MDI) [23].

The performance of a predictive model was evaluated using accuracy, sensitivity, specificity and area under the receiver operating characteristic (ROC) curve. Terminology and derivations of the metrics have been given in detail elsewhere [24]. Model success was defined as an area under the ROC curve (AUC) greater than 0.7 [25]. To find optimal hyperparameters during machine learning, K-fold cross-validation was used to train the models [26]. The dataset was split into 5 partitions, instantiated 5 identical models, and trained each one on 4 partitions while validating on the remaining partition. Then the average performance measures were calculated over the 5 folds. In the end, the choice of the model was the one with both a higher sensitivity and a higher specificity.

### Variable engineering

Because scalability is an important aspect of machine learning and matters for the models’ performance, variable engineering is preferred before training the models [27]. In total, there were 12 continuous variables (age, BMI, heart rate, systolic blood pressure, haemoglobin, c-reactive protein (CRP), creatinine, number of operations, physiology score, operative severity score, morbidity POSSUM, mortality POSSUM), two ordered variables (ASA classification and CCI) and 13 nominal variables (sex, cardiac condition, pulmonary condition, surgery indication, operation type, cancer, PRBC transfusion, osteopenia, postoperative infection requiring antibiotic treatment, heart failure, MI, arrhythmia, and kidney failure dialysis). Because of the extreme asymmetric distribution, CRP, creatinine, morbidity POSSUM and mortality POSSUM were log transformed before scaling. Since the aim of the current study was to predict the outcome, rather than to interpret the associations between the predictors and the outcome [22], therefore, all the variables were treated as continuous or discrete numerical numbers and were scaled using the Standard scaler to have the mean 0 and standard deviation 1. We also used other engineering methods such as dummy variables, and Robust and Min-Max scalers in sensitivity analysis.

### Variable importance

For the logistic regression model, the permutation variable importance (PVI) was calculated for each variable, which is measured by looking at how much the accuracy decreases when the information of a variable is not available [28]. To mask the information of a variable during training, instead of removing it from the dataset, the PVI method replaced it with random noise by shuffling its values from the patients. This is how the permutation works [29]. In the random forest model, the Gini importance was calculated for each variable, which was calculated as the sum over the number of splits (across all trees) that include the variable, proportionally to the number of samples in each split. The Gini importance indicates how often a particular variable was selected for a split and how large its overall discriminative value was for the classification problem under study [23, 30].

### Software and hardware

The descriptive and inferential statistical analyses were performed using Stata 15.1 (StataCorp LLC, College Station, TX, USA). The logistic regression and random forest models were achieved in Python 3.6 (Python Software Foundation, https://www.python.org/). All the computation was conducted on a computer with 64-bit Windows 7 Enterprise operating system (Service Pack 1), Intel® Core TM i5-4210U CPU @ 2.40 GHz, and 16.0 GB installed random access memory.

## Results

### Demographics and clinical outcomes of the patients

Originally 209 patients were included in the study. Fifty-two patients with missing values for any variable were excluded from the current study. In total, 157 patients with complete information were included in the final analysis with a mean age of 75.4 (standard deviation (SD) = 7.3) years. No statistically significant difference was found between the included and excluded patients (Table S1). There was an equal split between men and women. The average CCI score was 5.9 (SD = 2.3, median = 6.0, interquartile range (IQR): [4.0, 7.0]) and the most common ASA class was 3 (51.6%). The most common indication for surgery was bowel obstruction occurring in 92 (58.6%) patients. The most common surgical procedure during laparotomy was bowel resection with primary anastomosis occurring in 64 patients (40.8%). Types of post-operative complications ranged between 7.0–36.9% with infection being the most common type. Ninety-day mortality rate was 29.3% (Table 1). When comparing patients who died within 90 days of surgery to those who survived beyond this point, some statistically significant differences were detected. Patients who died within 90 days of surgery were on average older (mean age: 79 vs. 74 years, *p* < 0.001), had a higher average CCI (6.0 vs. 5.0, *p* = 0.001), a higher frequency of ASA class ≥4 (32.6% vs. 11.7%, p < 0.001), a lower mean systolic blood pressure prior at admission (123.2 vs. 132.1 mmHg, *p* = 0.022), higher proportions of PRBC transfusion requirements (58.7% vs. 30.6%, *p* = 0.002), osteopenia (63.0% vs. 36.0%, *p* = 0.003), and suffered more postoperative renal failure dialysis (15.2% vs. 4.5%, *p* = 0.049). Sarcopenia was only seen in less than 10% of patients and were for that reason left out from the analysis.

**Table 1:** Demographics and clinical outcome of the patients

Variables		All included patients (*n* = 157)	Alive after 90 days (*n* = 111, 70.7%)	Dead in 90 days (*n* = 46, 29.3%)	p*
Age (years), mean (SD)		75 (7)	74 (7)	79 (7)	< 0.001
Sex, n (%)	Female	78 (49.7)	54 (48.6)	24 (52.2)	0.821
	Male	79 (50.3)	57 (51.4)	22 (47.8)	
Cardiac condition, n (%)	No	56 (35.7)	42 (37.8)	14 (30.4)	0.485
	Yes	101 (64.3)	69 (62.2)	32 (69.6)	
Pulmonary condition, n (%)	No	97 (61.8)	70 (63.1)	27 (58.7)	0.740
	Yes	60 (38.2)	41 (36.9)	19 (41.3)	
Charlson index, mean (SD)		5.91 (2.30)	5.50 (2.12)	6.91 (2.44)	< 0.001
median [IQR]		6.00 [4.00, 7.00]	5.00 [4.00, 6.50]	6.00 [5.00, 8.75]	0.001
ASA class, n (%)	1	2 (1.3)	1 (0.9)	1 (2.2)	0.004
	2	46 (29.3)	40 (36.0)	6 (13.0)	
	3	81 (51.6)	57 (51.4)	24 (52.2)	
	4	27 (17.2)	13 (11.7)	14 (30.4)	
	5	1 (0.6)	0 (0.0)	1 (2.2)	
BMI (kg/m^2^), median [IQR]		24.5 [21.5, 27.1]	24.6 [21.3, 27.4]	24.4 [22.1, 26.0]	0.713
Heart rate (beats per minute), mean (SD)		87.01 (18.53)	86.71 (18.33)	87.74 (19.17)	0.753
Systolic blood pressure (mmHg), mean (SD)		129.48 (22.31)	132.10 (22.00)	123.17 (22.00)	0.022
Hemoglobin (g/L), mean (SD)		123.64 (22.45)	124.59 (22.32)	121.35 (22.82)	0.412
CRP (mg/L), median [IQR]		63.00 [20.00, 165.00]	58.00 [13.50, 172.00]	98.50 [48.50, 142.75]	0.100
Creatinine (μmol/L), median [IQR]		81.00 [64.00, 121.00]	76.00 [64.00, 114.50]	102.50 [65.25, 139.50]	0.064
Surgery indication, n (%)	Ileus/obstruction	92 (58.6)	67 (60.4)	25 (54.3)	0.266
	Perforation	37 (23.6)	25 (22.5)	12 (26.1)	
	Ischaemia	12 (7.6)	6 (5.4)	6 (13.0)	
	Infection	4 (2.5)	4 (3.6)	0 (0.0)	
	Bleeding	4 (2.5)	2 (1.8)	2 (4.3)	
	Other	8 (5.1)	7 (6.3)	1 (2.2)	
Number of operations, n (%)	1	109 (69.4)	79 (71.2)	30 (65.2)	0.761
	2	35 (22.3)	22 (19.8)	13 (28.3)	
	3	9 (5.7)	7 (6.3)	2 (4.3)	
	4	3 (1.9)	2 (1.8)	1 (2.2)	
	5	1 (0.6)	1 (0.9)	0 (0.0)	
Surgical procedure, n (%)	Resection with primary anastomosis	64 (40.8)	43 (38.7)	21 (45.7)	0.538
	Adhesiolysis	24 (15.3)	19 (17.1)	5 (10.9)	
	Resection with stoma formation	38 (24.2)	24 (21.6)	14 (30.4)	
	Other	18 (11.5)	15 (13.5)	3 (6.5)	
	Primary raphy	10 (6.4)	8 (7.2)	2 (4.3)	
	Embolectomy without bowel resection	3 (1.9)	2 (1.8)	1 (2.2)	
Cancer, n (%)	No	78 (49.7)	58 (52.3)	20 (43.5)	0.409
	Yes	79 (50.3)	53 (47.7)	26 (56.5)	
Blood Transfusion, n (%)	No	96 (61.1)	77 (69.4)	19 (41.3)	0.002
	Yes	61 (38.9)	34 (30.6)	27 (58.7)	
Physiology Score, mean (SD)		23.54 (6.56)	23.62 (5.98)	23.33 (7.86)	0.798
Operative Severity Score (points), mean (SD)		14.73 (2.93)	14.82 (2.79)	14.50 (3.26)	0.535
Morbidity POSSUM, median [IQR]		65.00 [48.80, 82.90]	66.40 [51.20, 82.90]	61.40 [43.08, 86.30]	0.322
Mortality POSSUM, median [IQR]		5.70 [2.80, 13.60]	5.70 [3.05, 12.95]	5.65 [2.50, 16.95]	0.518
Osteopenia, n (%)	No	88 (56.1)	71 (64.0)	17 (37.0)	0.003
	Yes	69 (43.9)	40 (36.0)	29 (63.0)	
Postoperative infection, n (%)	No	99 (63.1)	68 (61.3)	31 (67.4)	0.587
	Yes	58 (36.9)	43 (38.7)	15 (32.6)	
Postoperative heart failure, n (%)	No	147 (93.6)	105 (94.6)	42 (91.3)	0.682
	Yes	10 (6.4)	6 (5.4)	4 (8.7)	
Postoperative MI, n (%)	No	146 (93.0)	104 (93.7)	42 (91.3)	0.849
	Yes	11 (7.0)	7 (6.3)	4 (8.7)	
Postoperative arrhythmia, n (%)	No	125 (79.6)	93 (83.8)	32 (69.6)	0.073
	Yes	32 (20.4)	18 (16.2)	14 (30.4)	
Postoperative renal failure (dialysis), n (%)	No	145 (92.4)	106 (95.5)	39 (84.8)	0.049
	Yes	12 (7.6)	5 (4.5)	7 (15.2)	

^*^ Comparison between the alive and dead patients

ASA, American Society of Anaesthesiology; BMI, body mass index; CRP, C-reactive protein; IQR, interquartile range; SD, standard deviation; MI, myocardial infarction

### Performance of models

**Fig. 1:**
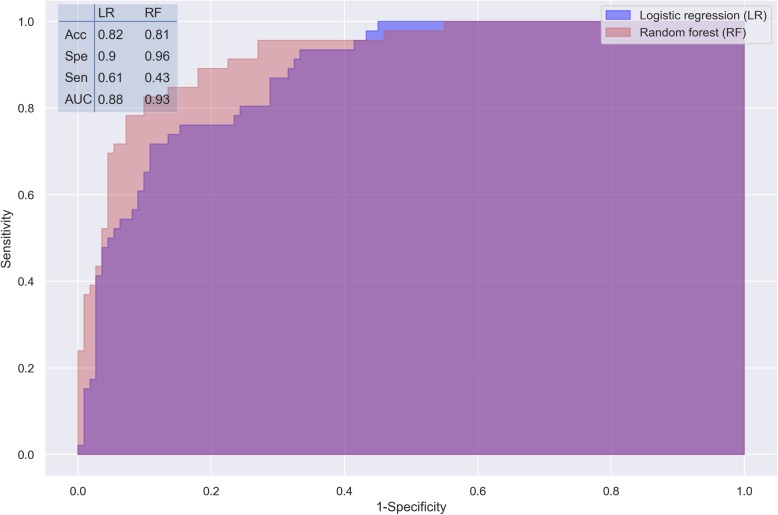
Performance measures of the logistic regression and random forest models, validated by K-fold cross-validation. Acc, accuracy; Spe, specificity; Sen, sensitivity; AUC, area under ROC curve

Both the logistic regression and random forest models using all the variables available showed improved performance for predicting 90-day mortality in geriatric patients after emergency laparotomy. The AUCs were 0.88 and 0.93 for logistic regression and random forest (Fig. 1), respectively. Both models had an accuracy > 0.8 and a specificity ≥0.9, which are acceptable in most clinical settings [31]. Although the random forest model had a greater AUC, its sensitivity was notably lower than that of the logistic regression model (0.43 vs. 0.61, Fig. 1).

### Importance of variables

**Fig. 2:**
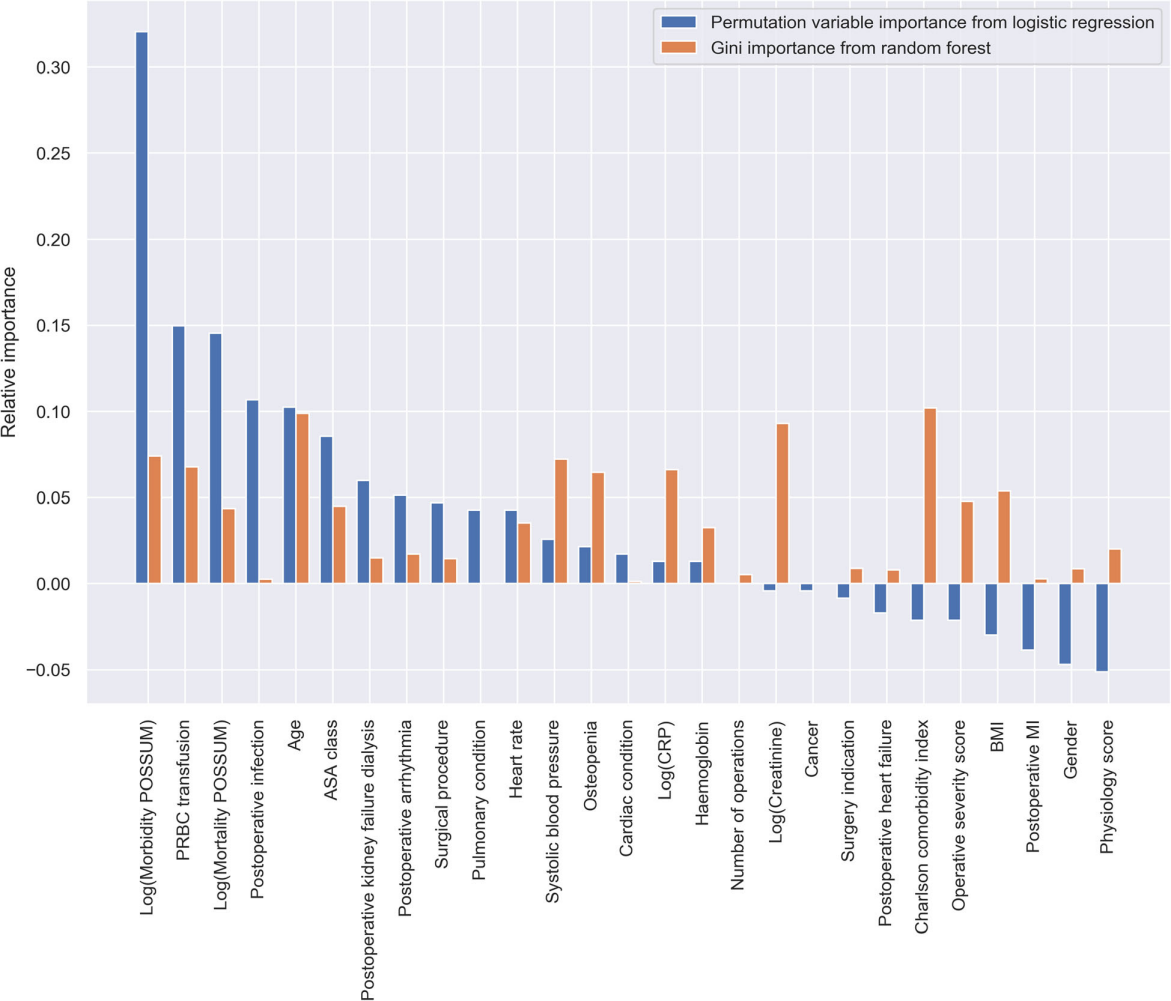
Relative variable importance of the logistic regression model and random forest model

The PVI derived from the logistic regression model indicated that morbidity P-POSSUM had the greatest relative importance for predicting 90-day mortality, followed by PRBC transfusion, mortality P-POSSUM, postoperative infection and age (Table 2 and Fig. 2). Considerable negative importance was observed for physiology score, sex, postoperative MI, BMI and operative severity score, indicating that these variables reduced the model’s predictive accuracy. According to the Gini importance derived from the random forest model, the top five variables with great importance are CCI, age, creatinine, systolic blood pressure, and morbidity P-POSSUM (Table 2 and Fig. 2).

**Table 2:** Top five important variables of the logistic regression model and random forest model

Rank	Permutation variable importance (PVI) of logistic regression model			Gini importance of random forest model		
	Standard scaler	Min-max scaler	Robust scaler	Standard scaler	Min-max scaler	Robust scaler
1	Morbidity POSSUM	Postoperative infection	Morbidity POSSUM	Charlson comorbidity index	Charlson comorbidity index	Charlson comorbidity index
2	PRBC transfusion	PRBC transfusion	Postoperative infection	Age	Age	Age
3	Mortality POSSUM	Age	PRBC transfusion	Creatinine	Creatinine	Creatinine
4	Age	Morbidity POSSUM	Mortality POSSUM	Systolic blood pressure	Systolic blood pressure	Systolic blood pressure
5	ASA class	Number of operations	Age	Morbidity POSSUM	Morbidity POSSUM	Morbidity POSSUM

ASA, American Society of Anaesthesiology; PRBC, packed red blood cell

### Sensitivity analysis

In sensitivity analysis, using dummy variables for nominal variables did not change or improve the predictive accuracy of the models, though the results of the logistic regression model would be easier to interpret clinically. When using Robust or Min-Max scalers instead of Standard scaler, the predictive accuracy of the logistic regression model was minimally reduced in sensitivity (Figs. S1 and S3), and the ranks of PVI also changed, however, morbidity P-POSSUM remained the first or the fourth important predictive variable (Figs. S2 and S4). The predictive accuracy and the top five important patient variables did not change irrespective of what scaler was used in the random forest model, and morbidity P-POSSUM was always one the five most important variables (Table 2 and Figs. S1–S4).

## Discussion

### Main findings

The aging population is increasing globally thus leading to more individuals being subjected to emergency surgery [32]. Emergency surgery in the geriatric patient has long been recognized as accruing excess morbidity and mortality than in younger patients, as a consequence of advanced age, increased burden of medical comorbidity and a loss of physiologic reserve [33]. This may limit the generalizability of existing predictive models and limit their utility in clinical planning and counselling the patients and their relatives. The ability to apply probabilistic methods to patient-level mortality prediction is important in informing surgical decision-making [34]. The identification of factors to predict outcomes after emergency laparotomy in this patient population is of paramount importance both in organizing healthcare systems and in clinical decision making, and communication with patients and their family members.

P-POSSUM is widely used for operative mortality prediction, however, its applicability in geriatric patients undergoing emergency laparotomy is still under debate. In our previous study, based on a Poisson regression analysis, we found that P-POSSUM alone had poor prognostic value in geriatric patients subjected to emergency laparotomy with an AUC of only 0.59 [22]. However, as observed in our previous study the P-POSSUM with adjustment for other patient and perioperative characteristics may improve its prediction for mortality in this patient population. Although standardized regression coefficients may partially reflect the relative importance of clinically available patient variables for predicting mortality, they cannot reflect the variables’ influence on the accuracy of prediction, because the predicted outcome is at a patient level, while accuracy of any generalized model describes probability at a population level [35]. In the current study, we further investigated the relative importance of P-POSSUM compared to other clinical risk factors for outcome prediction using machine learning methods. Several of the variables were indeed common in the models, i.e. age and cancer. However, it is important to notice that in the calculation of P-POSSUM, age is not a continuous variable, but is categorically binned; thus, for the geriatric population over 70 years, all patients are included in the same age group, leading to loss of discrimination between older and younger geriatric patients. This is an important distinction to make. Looking at 30-day mortality after emergency laparotomy using the National Emergency Laparotomy Audit (NELA) database, Eugene et al. detected an increase in the incidence of deaths per decade after 60 years of age from 9.9, 15.3, 20.2 to 24.2% [10]. Al-Temimi and colleagues analyzed 37,553 patients subjected to emergency laparotomy using the American College of Surgeons National Surgical Quality Improvement Program database. After adjustment they found an increased mortality risk with an odds ratio of 2.3, 3.5, 5.9 and 7.9 for the age groups 60–69 years, 70–79 years, 80–89 years and over 90 years, respectively [4].

Recognizing the unavoidable collinearity of constituent variables of P-POSSUM with their overall composite, our study suggests that while P-POSSUM is generally predictive of mortality in elderly patients undergoing emergency laparotomy, it under-represents the importance of age in the geriatric population, comorbidity and the effect of transfusion-dependent hemorrhage or anemia in accurately predicting outcomes in this population. More importantly, P-POSSUM morbidity is more predictive of mortality than the mortality calculator in the geriatric population undergoing emergent laparotomy, which would imply a re-calibration of the model for the geriatric age range should be considered. Previous studies have shown that morbidity, i.e. complication, is significantly associated with both short- and long-term survival after surgery [36]. This adverse outcome was also detected in patients subjected to emergency laparotomy for colon cancer who had a post-operative complication [37].

### Predictive model selection

There are other machine learning algorithms available, such as discriminant analysis, decision tree, K-nearest neighbor, support vector machine, and multilayer perceptron, for supervised classification problems [24]. The reasons for using and comparing logistic regression to random forest in the current study are:

a) Logistic regression is the most widely used method in diagnostic tests and prediction studies for binary outcomes in medical sciences. The results from logistic regression analysis can be easily comprehended by clinical researchers [38–40]. Coefficients from the logistic regression models can be translated into odds ratios, which are widely used in medical and epidemiology studies. All the five top important variables (morbidity P-POSSUM, PRBC transfusion, mortality P-POSSUM, age and ASA class) found by the logistic regression model in the current study are consistent with the statistically significant risk factors derived from the previously reported stepwise Poisson regression analysis [22].

b) Random forest is an ensemble method in which the classifier is constructed by combining several different independent base classifiers [41]. Although its application is a bit limited in medical and life sciences because of its complexity and more computational cost, it has several advantages over basic machine learning methods, including reduction in overfitting and less variance [30, 42–44]. In general, we can see that the random forest model has higher accuracy than the logistic regression model, however, its sensitivity is lower (Fig. 1, S1, and S3).

### Variable selection and variable importance

Variable selection is one of the core concepts in machine learning which hugely impacts the performance of predictive models. Irrelevant or partially relevant variables can negatively impact model performance, just as the ones with negative PVI that we observed in Fig. 2, S2 and S4. There are many variable selection methods available in data science, such as recursive variable elimination, principle component analysis, correlation matrix with heatmap, variable importance, and some wrapper methods [45, 46], and variable importance is a straightforward one that can be easily explained to the audience out of the fields of data science. In the current study, we calculated and compared two kinds of variable importance, i.e. the PVI and the Gini importance. Compared to Gini importance, which is embedded in tree based machine learning algorithms such as random forest, the PVI method is a model-agnostic approach, which permutes the values of a variable of interest and reevaluate model performance [28]. The observed accuracy decrease in performance indicates variable importance. The method is generalizable no matter the predictive model and most suitable for computing variable importance when the number of variables is not huge, otherwise it can be resource-intensive [28, 29]. We cannot compare the PVI and Gini importance directly, because they were calculated based on different rationales. However, we may compare the ranks that they reflect, which may be useful when we want to find common important variables in different machine learning methods.

### Variable scaling

Variable engineering is an important step for machine learning in data science. Although variable scaling (or variable standardization in medical and epidemiology studies) methods such as standardization have been used in medical studies for a long time [47], they are sometimes overlooked in regression analysis and results interpretation, where researchers are more interested in explaining the association between the risk factors and outcomes rather than the accuracy of the prediction, which is seldom evaluated in clinical and epidemiology studies other than in diagnostic tests [48, 49]. In the current study, we compared three scalers in both the logistic regression model and the random forest model, and obtained different importance ranks for the logistic regression model. The results suggest that we need to take the scaling method into account when evaluate the importance or contribution of the variables to the prediction.

### Strengths and limitations

There are several strengths in the current study. To the best of our knowledge, this is the first study investigating the predictive rather than associative value of P-POSSUM and other patient, operative and postoperative characteristics with mortality in geriatric patients undergoing emergency laparotomy. Secondly, our data suggest that not only is P-POSSUM generally applicable to prediction exercises in our geriatric population, but the addition of age for the geriatric age range, comorbidity and the physiologic response to hemorrhage or anemia requiring blood transfusions further improve the precision and accuracy of the model output. This has tangible clinical benefits both in informing clinical decision-making and in translating statistical probability into coherent information for elderly patients and their family. Thus, a consensus plan (either operative or palliative) may be more readily reached. Thirdly, cross-validation was used when we built the models. The hyperparameters for machine learning were tuned according to the average accuracy of five validations rather than a single model to avoid overfitting. Finally, two variable importance methods and three scaling methods were applied in our data analysis. Therefore, six importance measures in total were calculated for each variable, which may depict the variance of their importance, and ensure our conclusion being conservative and robust.

However, there are also several potential limitations in the study. A slightly higher rate of mortality incidence was detected in our studied cohort compared with previous studies from European countries with similar healthcare. Saunders and colleagues reported a 24.4% 30-day mortality in the same age group as ours who had undergone an emergency laparotomy [2]. The 90-day mortality for patients over 80 years subjected to laparotomy was 25.2% reported by Simpson et al. [3] One explanation to this finding could be the selection of patients to laparotomy at our center, where in most cases a laparoscopic approach is preferred in patients who are deemed more stable or when a less complex surgery is expected. The exclusion of conversion from a laparoscopic approach to laparotomy might also introduce a bias including a higher proportion of more severely ill patients in the current study. This is demonstrated by the fact that patient who did not survive beyond 90-day post-laparotomy were older with higher incidence of osteopenia, higher ASA classification, more hypotensive at admission, and in need of more blood transfusions. Further, only 157 patients were included in our cohort. Essentially, the performance of machine learning methods relies on the amount of data available. The more data, the better the models perform. Although we obtained satisfactory accuracy from both models, the generalizability and external validity of our model are limited by the small sample size. More data are needed for model training in the future. Second, nominal variables were treated as discrete numbers in the study. Although it increases the accuracy of prediction, the interpretability of the models was reduced. Third, patients with missing values were excluded from analysis. We tried to include these patients by using the multiple imputation method, however, the accuracy of prediction was reduced as a result. Although multiple imputation may help to provide more robust estimates for inferential statistical analysis, it seems to introduce noise rather than information for prediction. Assigning the missing values as a unique category or using algorithms such as k-nearest neighbors that support values are deserved investigation in the future.

## Conclusion

P-POSSUM is important for predicting 90-day mortality after emergency laparotomy in geriatric patients. The logistic regression model and random forest model may have an accuracy of > 0.8 and an AUC around 0.9 for predicting 90-day mortality. Further validation of the variables’ importance and the models’ robustness is needed by use of larger dataset.

## Supplementary information

**Additional file 1: Table S1.** Demographics and clinical outcome of the included and excluded patients. **Figure S1.** Performance measures of the logistic regression and random forest models, with data transformed by Min-Max scaler. Acc, accuracy; Spe, specificity; Sen, sensitivity; AUC, area under ROC curve. **Figure S2.** Relative variable importance of logistic regression and random forest models, with data transformed by Min-Max scaler. **Figure S3.** Performance measures of the logistic regression and random forest models, with data transformed by Robust scaler. Acc, accuracy; Spe, specificity; Sen, sensitivity; AUC, area under ROC curve. **Figure S4.** Relative variable importance of logistic regression and random forest models, with data transformed by Robust scaler.

## Data Availability

The database for the current study was created and analyzed after approval of IRB which stated that only the authors are allowed access to the data. The authors would ask for further IRB permission to release an unidentifiable dataset to the Editorial Board on request.

## Abbreviations

APACHE: Acute Physiology and Chronic Health Evaluation
ASA: American Society of Anaesthesiology
AUC: area under the receiver operating characteristic curve (AUC)
BMI: Body mass index
CCI: Charlson Comorbidity Index
CRP: C-reactive protein
CT: Computed tomography
ICD: International Statistical Classification of Diseases
IQR: Interquartile range
LR: Logistic regression
MDI: mean decrease in impurity
MI: Myocardial infarction
P-POSSUM: Portsmouth - Physiological and Operative Severity Score for the enumeration of Mortality and morbidity
PVI: Permutation variable importance
PMP: Pre-operative Mortality Predictor
PRBC: Packed red blood cell
RF: Random forest
ROC: Receiver operating characteristic
SD: Standard deviation
SRS: Surgical Risk Scale
