# Supplementary material for: The statistical importance of P-POSSUM scores for predicting mortality after emergency laparotomy in geriatric patients
Source: BMC Med Inform Decis Mak. 2020 May 7;20:86. doi: 10.1186/s12911-020-1100-9 (PMC7206787; doi:10.1186/s12911-020-1100-9)
Supplement: Supplementary file 1 — Additional file 1: Table S1. Demographics and clinical outcome of the included and excluded patients. Figure S1. Performance measures of the logistic regression and random forest models, with data transformed by Min-Max scaler. Acc, accuracy; Spe, specificity; Sen, sensitivity; AUC, area under ROC curve. Figure S2. Relative variable importance of logistic regression and random forest models, with data transformed by Min-Max scaler. Figure S3. Performance measures of the logistic regression and random forest models, with data transformed by Robust scaler. Acc, accuracy; Spe, specificity; Sen, sensitivity; AUC, area under ROC curve. Figure S4. Relative variable importance of logistic regression and random forest models, with data transformed by Robust scaler. [file 12911_2020_1100_MOESM1_ESM.docx]

**Supplementary materials**

**Table S1.** Demographics and clinical outcome split of the included and excluded patients

| Variables |  | All patients  (n=209) | Included  (n=157) | Excluded  (n=52) | p^*^ |
| --- | --- | --- | --- | --- | --- |
| Ninety-day mortality, n (%) | Alive | 144 (68.9) | 111 (70.7) | 33 (63.5) | 0.421 |
|  | Dead | 65 (31.1) | 46 (29.3) | 19 (36.5) |  |
| Sex, n (%) | Female | 104 (49.8) | 78 (49.7) | 26 (50.0) | 1.000 |
|  | Male | 105 (50.2) | 79 (50.3) | 26 (50.0) |  |
| Age (years), mean (SD) |  | 75.58 (7.29) | 75.43 (7.12) | 76.04 (7.82) | 0.601 |
| Cardiac condition, n (%) | No | 83 (39.7) | 56 (35.7) | 27 (51.9) | 0.056 |
|  | Yes | 126 (60.3) | 101 (64.3) | 25 (48.1) |  |
| Pulmonary condition, n (%) | No | 136 (65.1) | 97 (61.8) | 39 (75.0) | 0.118 |
|  | Yes | 73 (34.9) | 60 (38.2) | 13 (25.0) |  |
| Charlson index, mean (SD) |  | 5.77 (2.36) | 5.91 (2.30) | 5.35 (2.50) | 0.135 |
| median [IQR] |  | 6.00 [4.00, 7.00] | 6.00 [4.00, 7.00] | 5.00 [3.00, 6.25] | 0.124 |
| ASA class, n (%) | 1 | 4 ( 2.0) | 2 ( 1.3) | 2 ( 4.8) | 0.092 |
|  | 2 | 54 (27.1) | 46 (29.3) | 8 (19.0) |  |
|  | 3 | 106 (53.3) | 81 (51.6) | 25 (59.5) |  |
|  | 4 | 32 (16.1) | 27 (17.2) | 5 (11.9) |  |
|  | 5 | 3 ( 1.5) | 1 ( 0.6) | 2 ( 4.8) |  |
| BMI (kg/m^2^), median [IQR] |  | 24.5 [21.5, 27.3] | 24.5 [21.5, 27.1] | 24.1 [20.8, 28.6] | 0.702 |
| Heart rate (beats per minute), mean (SD) |  | 85.75 (18.37) | 87.01 (18.53) | 81.94 (17.51) | 0.084 |
| Systolic blood pressure (mmHg), mean (SD) |  | 129.96 (23.16) | 129.48 (22.31) | 131.40 (25.75) | 0.606 |
| Hemoglobin (g/L), mean (SD) |  | 124.04 (23.66) | 123.64 (22.45) | 125.35 (27.43) | 0.660 |
| CRP (mg/L), median [IQR] |  | 63.00 [17.50, 178.00] | 63.00 [20.00, 165.00] | 60.50 [10.43, 218.50] | 0.774 |
| Creatinine (µmol/L), median [IQR] |  | 85.00 [68.00, 125.00] | 81.00 [64.00, 121.00] | 95.00 [72.25, 131.00] | 0.069 |
| Surgery indication, n (%) | Ileus/obstruction | 119 (56.9) | 92 (58.6) | 27 (51.9) | 0.219 |
|  | Perforation | 45 (21.5) | 37 (23.6) | 8 (15.4) |  |
|  | Ischaemia | 19 ( 9.1) | 12 ( 7.6) | 7 (13.5) |  |
|  | Infection | 5 ( 2.4) | 4 ( 2.5) | 1 ( 1.9) |  |
|  | Bleeding | 8 ( 3.8) | 4 ( 2.5) | 4 ( 7.7) |  |
|  | Other | 13 ( 6.2) | 8 ( 5.1) | 5 ( 9.6) |  |
| Number of operations, n (%) | 1 | 141 (67.8) | 109 (69.4) | 32 (62.7) | 0.754 |
|  | 2 | 47 (22.6) | 35 (22.3) | 12 (23.5) |  |
|  | 3 | 13 ( 6.2) | 9 ( 5.7) | 4 ( 7.8) |  |
|  | 4 | 5 ( 2.4) | 3 ( 1.9) | 2 ( 3.9) |  |
|  | 5 | 2 ( 1.0) | 1 ( 0.6) | 1 ( 2.0) |  |
| Surgical procedure, n (%) | Resection with primary anastomosis | 82 (39.2) | 64 (40.8) | 18 (34.6) | 0.428 |
|  | Adhesiolysis | 38 (18.2) | 24 (15.3) | 14 (26.9) |  |
|  | Resection with stoma formation | 46 (22.0) | 38 (24.2) | 8 (15.4) |  |
|  | Other | 25 (12.0) | 18 (11.5) | 7 (13.5) |  |
|  | Primary raphy | 14 ( 6.7) | 10 ( 6.4) | 4 ( 7.7) |  |
|  | Embolectomy without bowel resection | 4 ( 1.9) | 3 ( 1.9) | 1 ( 1.9) |  |
| Cancer, n (%) | 0 | 111 (53.1) | 78 (49.7) | 33 (63.5) | 0.117 |
|  | 1 | 98 (46.9) | 79 (50.3) | 19 (36.5) |  |
| Blood transfusion, n (%) | No | 124 (59.3) | 96 (61.1) | 28 (53.8) | 0.444 |
|  | Yes | 85 (40.7) | 61 (38.9) | 24 (46.2) |  |
| Physiology Score, mean (SD) |  | 23.65 (6.64) | 23.54 (6.56) | 24.00 (6.93) | 0.665 |
| Operative Severity Score (points), mean (SD) |  | 14.73 (2.91) | 14.73 (2.93) | 14.75 (2.88) | 0.968 |
| Morbidity POSSUM, median [IQR] |  | 66.60 [48.00, 84.23] | 65.00 [48.80, 82.90] | 76.30 [44.05, 85.85] | 0.319 |
| Mortality POSSUM, median [IQR] |  | 6.55 [2.80, 15.55] | 5.70 [2.80, 13.60] | 9.70 [2.50, 17.75] | 0.328 |
| Osteopenia, n (%) | 0 | 104 (53.3) | 88 (56.1) | 16 (42.1) | 0.172 |
|  | 1 | 91 (46.7) | 69 (43.9) | 22 (57.9) |  |
| Postoperative infection, n (%) | No | 133 (63.9) | 99 (63.1) | 34 (66.7) | 0.765 |
|  | Yes | 75 (36.1) | 58 (36.9) | 17 (33.3) |  |
| Postoperative heart failure, n (%) | No | 198 (94.7) | 147 (93.6) | 51 (98.1) | 0.375 |
|  | Yes | 11 ( 5.3) | 10 ( 6.4) | 1 ( 1.9) |  |
| Postoperative MI, n (%) | No | 197 (94.3) | 146 (93.0) | 51 (98.1) | 0.307 |
|  | Yes | 12 ( 5.7) | 11 ( 7.0) | 1 ( 1.9) |  |
| Postoperative arrhythmia, n (%) | No | 165 (78.9) | 125 (79.6) | 40 (76.9) | 0.828 |
|  | Yes | 44 (21.1) | 32 (20.4) | 12 (23.1) |  |
| Postoperative renal failure (dialysis), n (%) | No | 191 (91.4) | 145 (92.4) | 46 (88.5) | 0.560 |
|  | Yes | 18 ( 8.6) | 12 ( 7.6) | 6 (11.5) |  |

^*^ Comparison between the included and excluded patients.

ASA, American Society of Anaesthesiology; BMI, body mass index; CRP, C-reactive protein; IQR, interquartile range; SD, standard deviation; MI, myocardial infarction


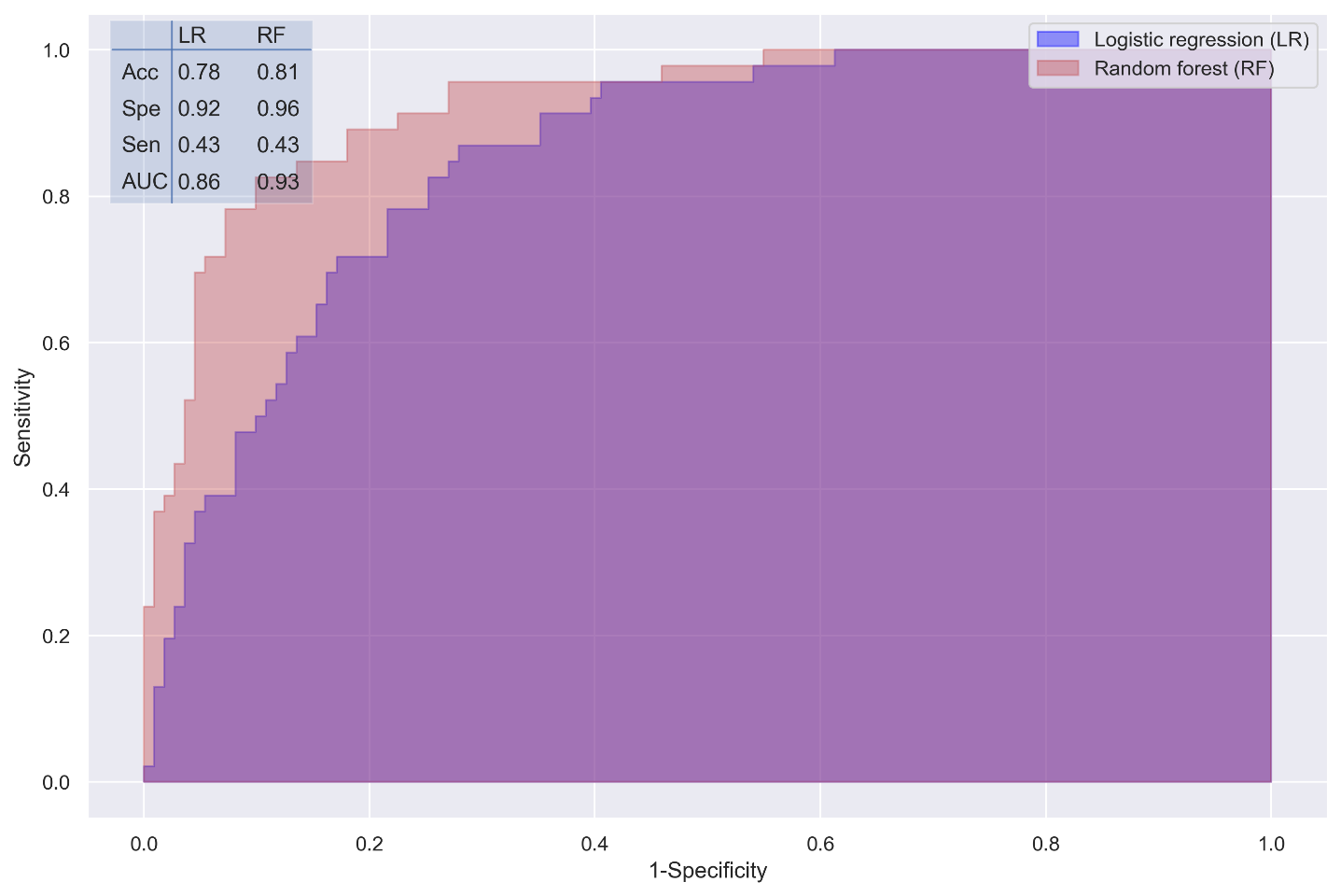


**Figure S1.** Performance measures of the logistic regression and random forest models, with data transformed by Min-Max scaler. Acc, accuracy; Spe, specificity; Sen, sensitivity; AUC, area under ROC curve


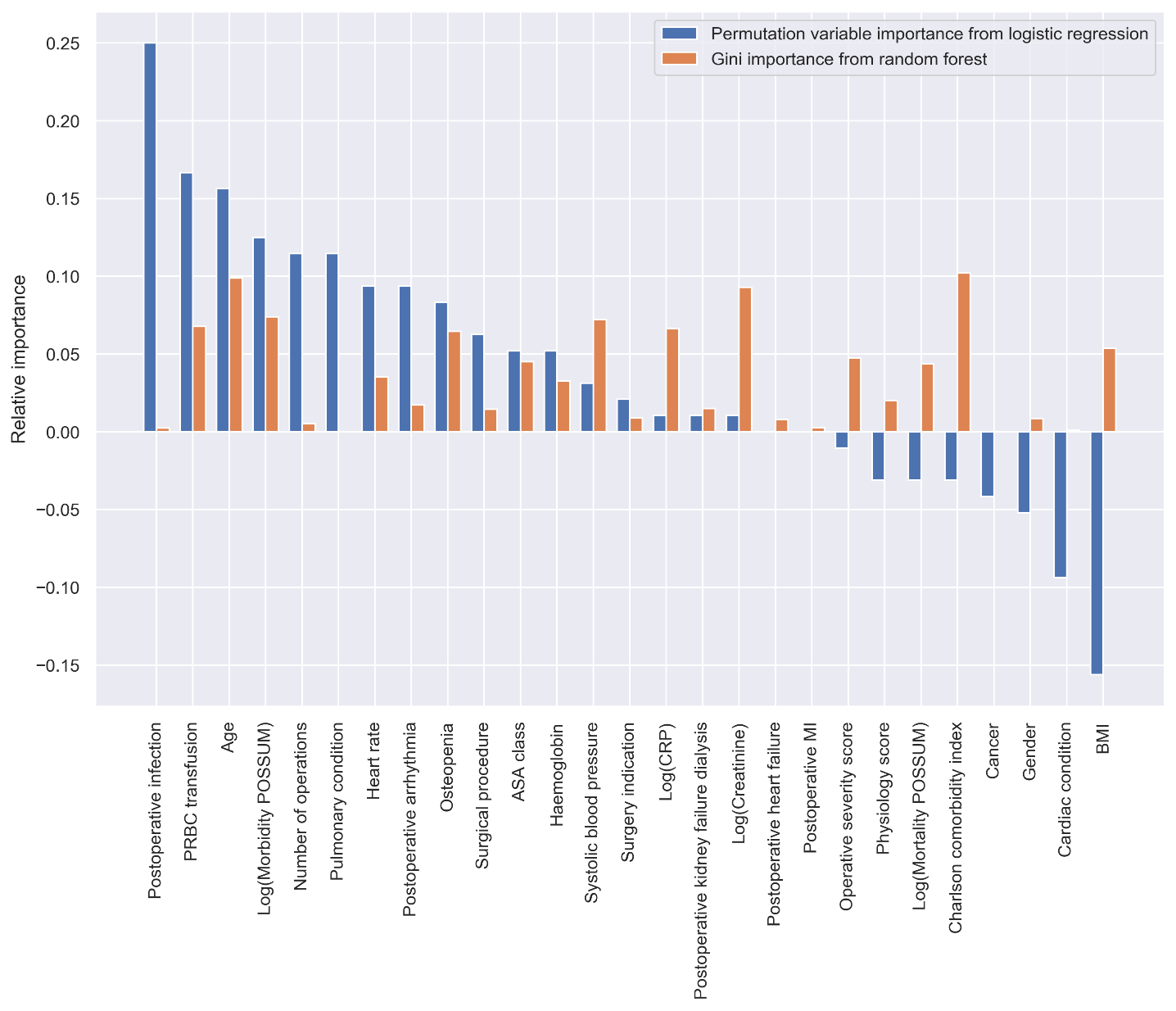


**Figure S2.** Relative variable importance of logistic regression and random forest models, with data transformed by Min-Max scaler


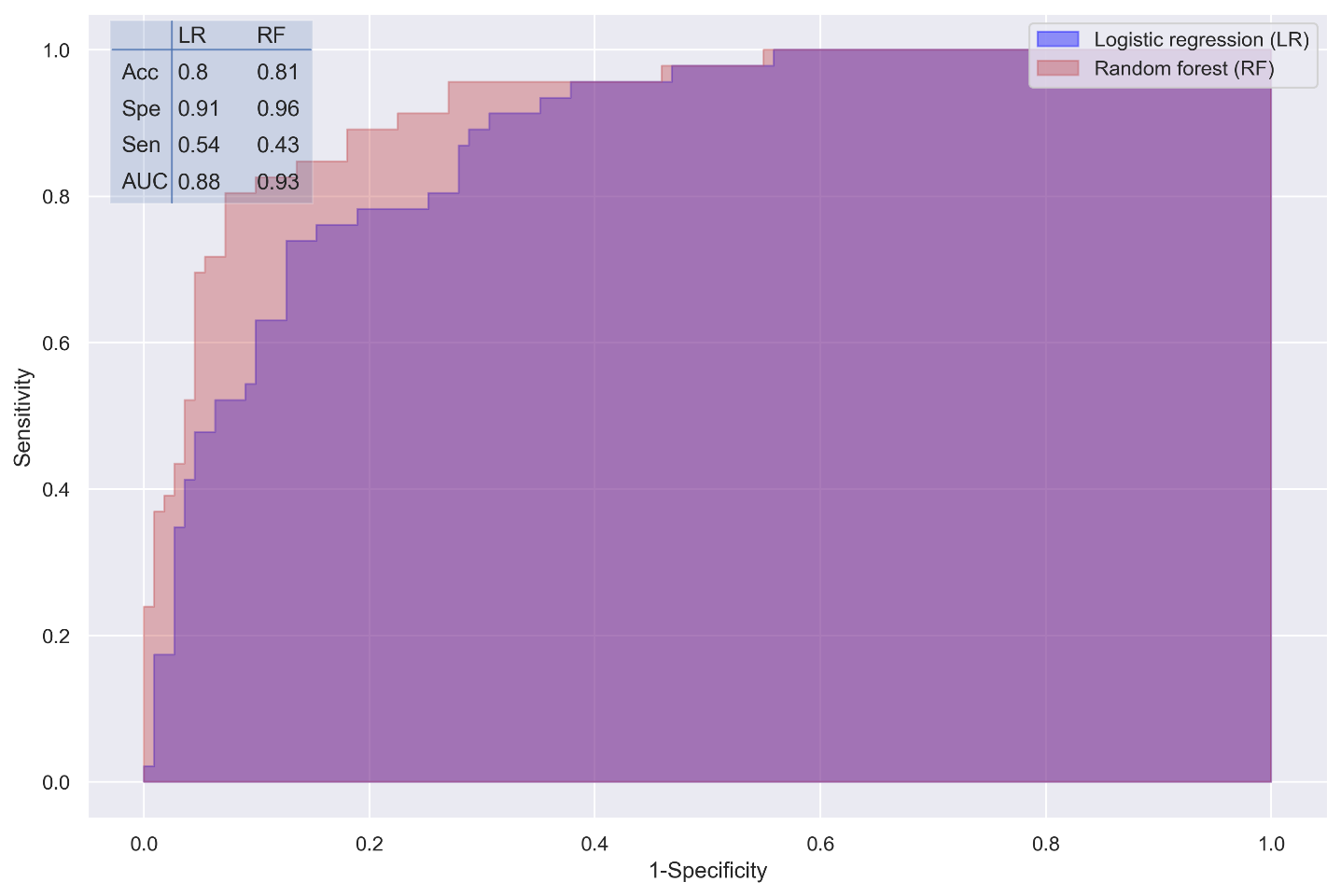


**Figure S3.** Performance measures of the logistic regression and random forest models, with data transformed by Robust scaler. Acc, accuracy; Spe, specificity; Sen, sensitivity; AUC, area under ROC curve


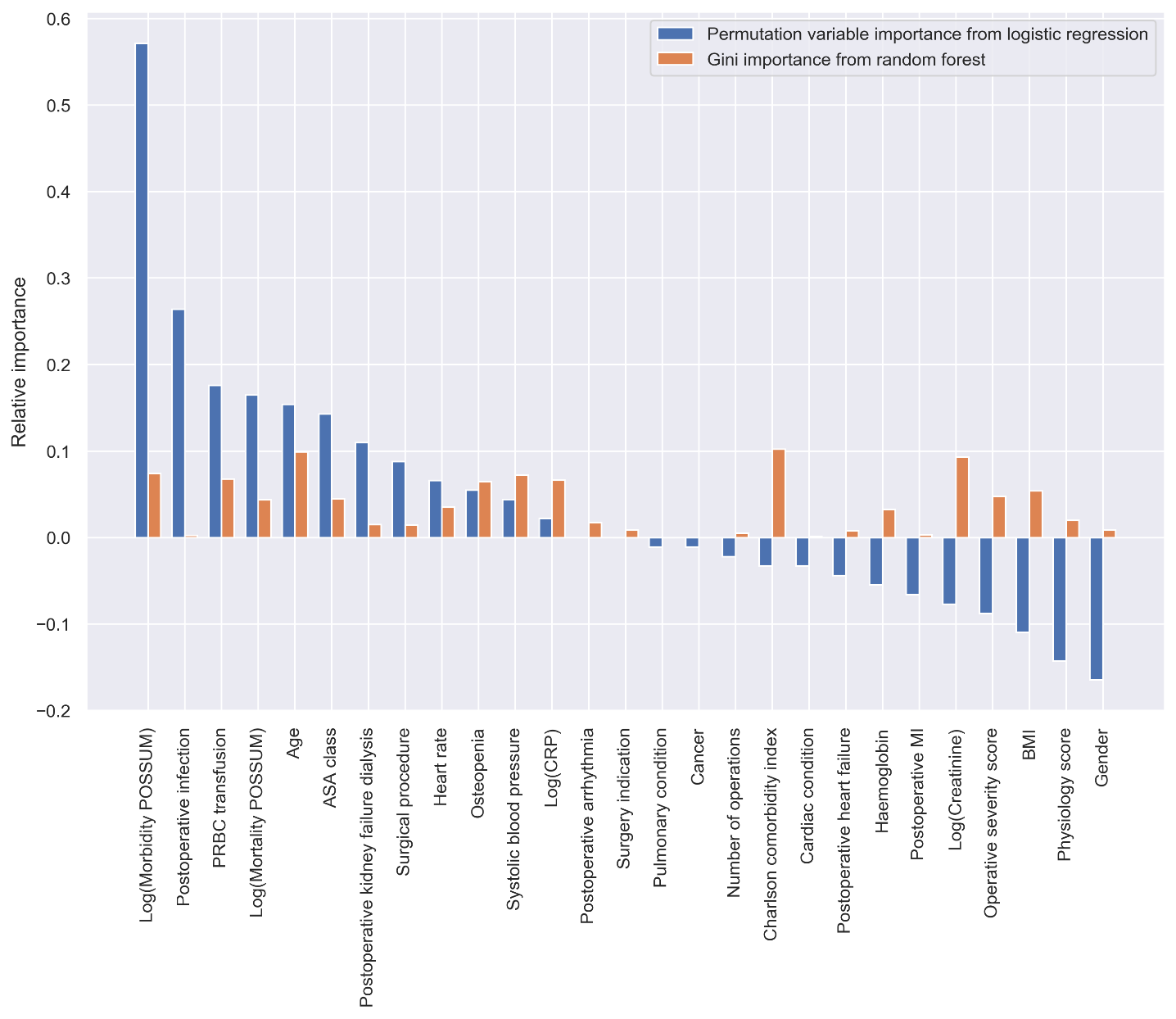


**Figure S4.** Relative variable importance of logistic regression and random forest models, with data transformed by Robust scaler
